# Current breast cancer proliferative markers correlate variably based on decoupled duration of cell cycle phases

**DOI:** 10.1038/srep05122

**Published:** 2014-05-30

**Authors:** Lik Hang Lee, Hua Yang, Gilbert Bigras

**Affiliations:** 1 Department of Laboratory Medicine and Pathology, University of Calgary, AB, Canada; 2 Department of Laboratory Medicine and Pathology, Cross Cancer Institute, University of Alberta, AB, Canada

## Abstract

Mitotic count, PhH3, and MIB-1 are used as measures of the proportion of proliferating malignant cells in surgical pathology. They highlight different stages of the cell cycle, but little is known about how this affects their counts. This study assesses the strength of their correlations and attempts to determine the relationship between them. Proliferation counts for forty-nine consecutive cases of invasive breast carcinomas were analyzed, with the same tumor area on each stain counted using digital image analysis. The integrated optical density (IOD) of nuclei was measured as an approximation of nuclear DNA content. PhH3 strongly correlated with mitotic count (r = 0.94). Weaker correlations were found between MIB-1 versus PhH3 (r = 0.79) and mitotic count (r = 0.83). Nuclear IOD showed stronger correlation with MIB-1 (r = 0.37) than to mitotic count (r = 0.23) and PhH3 (r = 0.34). With evidence from a literature review, it is suggested that the weaker correlations with MIB-1 are not explained by count imprecision or error, but relies on temporal decorrelation between cell cycle phases. Consequences on correlation between these proliferative markers are illustrated by mathematical models.

The proportion of malignant cells undergoing proliferation has become an important variable in diagnostic surgical pathology, particularly in malignant neoplasms. This variable significantly influences tumor growth rate, and thus, represent tumoral aggressiveness. It is used to assess the malignant potential, such as the grade, of many neoplasms. More recently, it has been playing an increasingly important role in its predictive ability to guide treatment, such as in breast carcinoma^1^,^2^,^3^,^4^,^5^.

There have been many methods of quantifying the proportion of proliferating cells. The traditional mitotic count is still used to grade a variety of tumors including breast adenocarcinomas, neuroendocrine tumors, sarcomas, meningiomas, and melanomas^6^,^7^,^8^,^9^,^10^,^11^. It is important to remember that the proportion of proliferating cells provides incomplete information about tumor growth; the net amount of malignant cells at a certain time is the balance between the rate of newly produced cells and the rate of cell death from necrosis and apoptosis. Neglecting the negative impact of cell death, the total number of malignant cells at a given point in time is 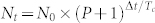 where N_0_ is the initial number of cells, Δ*t* is a given interval of time, T_c_ is the cell cycle duration, and P is the fraction of proliferating cells. Most known proliferative markers only attempt to assess P. Cell death is generally assessed only as a nominal variable (present versus absent) because there is no quantitative model to measure it. In addition to the mitotic count, immunohistochemical markers of proliferation have been more recently developed and include MIB-1 which is directed at the Ki-67 antigen and targets the whole cell cycle^2^,^12^, proliferating cell nuclear antigen (PCNA) which targets the S phase^13^, and phosphorylated histone H3 (PhH3) which targets the mitotic phase^14^.

While new immunohistochemical markers have been developed, mitosis remains the most important proliferative marker in clinical breast pathology. Elston and Ellis showed that if breast cancer grading protocol, which includes the mitotic score, is followed consistently, reproducible results can be obtained for breast tumor prognosis^9^. However mitotic score assessment is time consuming, and is not consistent due to problems of reproducibility, even amongst trained pathologists^15^,^16^,^17^.

MIB-1 would make the visualization of proliferating cells easier^16^. It stains all nuclei involved in the cell cycle (G1, S, G2, and mitoses)^2^ and has emerged as an important independent prognostic and predictive marker in several tumors, including breast cancer^1^, sarcomas^16^,^18^, cutaneous melanomas^11^,^19^,^20^, meningiomas^21^, and prostate cancer^22^ among others. More specifically for breast cancer, the level of MIB-1 expression prior to neoadjuvant chemotherapy is a strong predictive factor for the potential effectiveness of the therapy, and post-therapy expression predicts disease-free survival^1^,^3^,^4^,^5^. The St. Gallen Consensus Statements on breast cancer have supported the use of the Ki-67 index (as determined by MIB-1) in deciding whether to offer chemotherapy to patients with hormone positive, node negative cancers, and also as part of the criteria in separating luminal A from luminal B carcinomas^23^,^24^. As a result, the Ki-67 index is increasingly being requested by medical oncologists to guide their breast cancer treatment decisions. While the function of the Ki-67 protein remains unknown, there is evidence that it has a role in cell division and ribosomal RNA synthesis^25^,^26^. The protein name is non-descriptive, named after Kiel University in Germany where it was discovered with 67 referring to the clone number^27^.

PhH3, in contrast to MIB-1, targets cells in the mitotic phase. Histone H3 is a nuclear core histone protein that is a constituent of chromatin. Its phosphorylation at serine-10 and serine-28 are believed to be crucial for chromosome condensation and cell-cycle progression during mitosis and meiosis^28^,^29^,^30^. Phosphorylation initiates in late G2 phase to early prophase and gradual dephosphorylation occurs from late anaphase to early telophase. Metaphase chromosomes are always heavily phosphorylated while interphase cells do not stain or only do so with low intensity^14^,^29^,^30^,^31^,^32^. Thus, PhH3 counts should theoretically correlate with mitotic counts and has emerged as a potential immunohistochemical marker of mitotic activity. Indeed several reports showing positive correlation between mitotic and PhH3 counts have been published^20^,^21^,^33^,^34^,^35^,^36^,^37^.

Few studies have compared MIB-1 with PhH3^20^,^21^,^33^,^34^,^38^,^39^,^40^. Considerable variability in correlation between MIB-1 counts and PhH3 counts are reported, but they used methodologies that do not address the intratumoral heterogeneity observed in most solid malignant tumors which would reduce correlation between markers^2^,^41^. The aim of this study was to measure the correlation between MIB-1 and PhH3 labeling, as well as mitotic counts, on breast adenocarcinoma. The same tumor area on each stain was assessed in order to eliminate the influence of intratumoral heterogeneity. Even if each marker assesses different intervals of the cell cycle, these markers all measure the fraction of proliferating cells and would thus be expected to strongly correlate, particularly if the durations of these intervals remain proportionally constant. Correlations with MIB-1 and DNA content (approximated by the nuclear integrated optical density (IOD)) were assessed in order to further understand the relationships among all variables.

## Results

**Figure 1:**
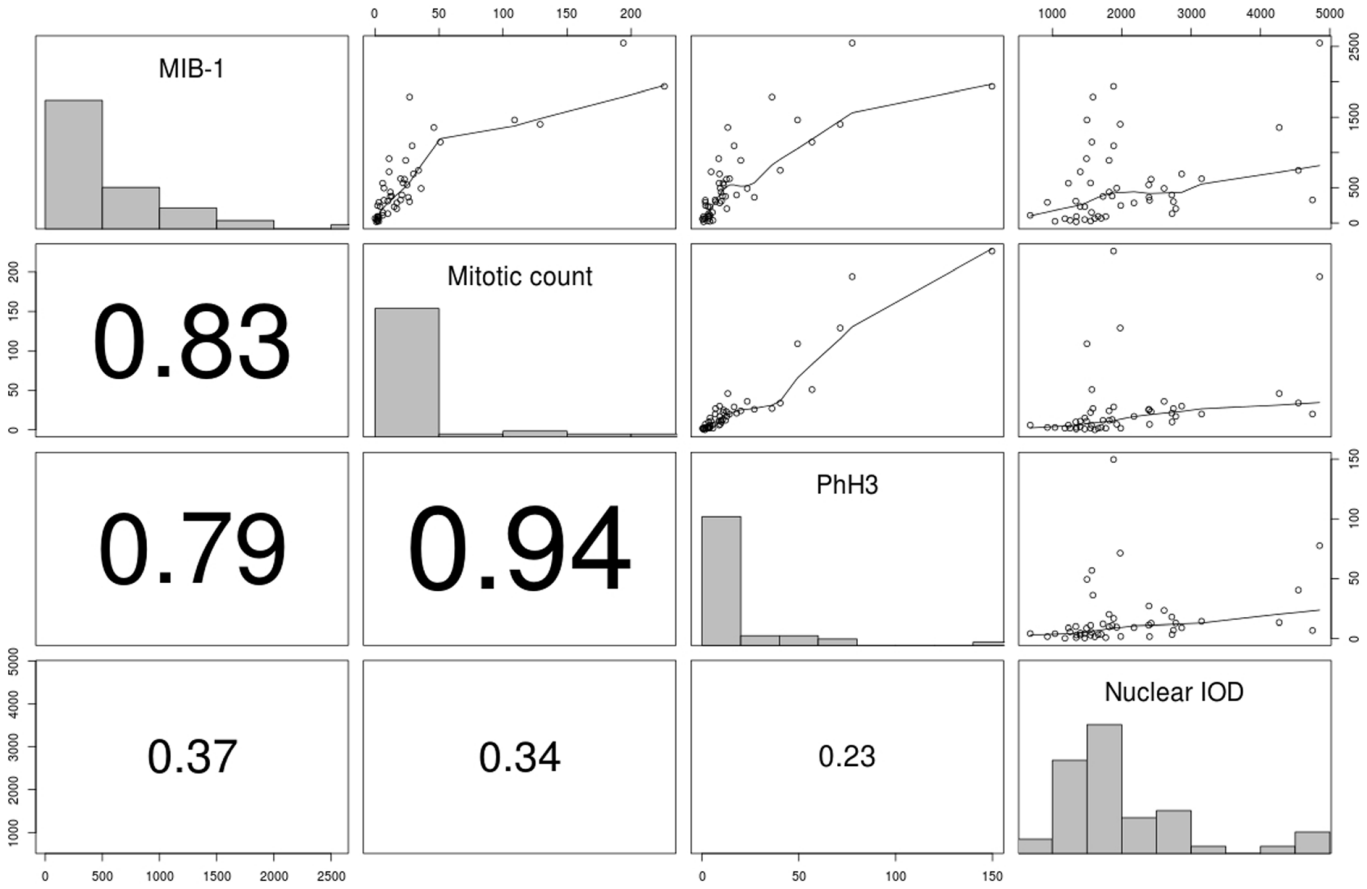
Scatterplot matrix for continuous variables (mitotic count, MIB-1, PhH3 and Nuclear IOD). Variables are crossed against each other (upper right). In each scatterplot a black line illustrates average trend. The diagonal shows histogram of each variable. Correlation coefficients (Pearson) are found in lower left. The strongest correlation (0.94) is found between mitotic count and PhH3. The strongest correlation value involving nuclear IOD is 0.37 with MIB-1.

**Figure 2:**
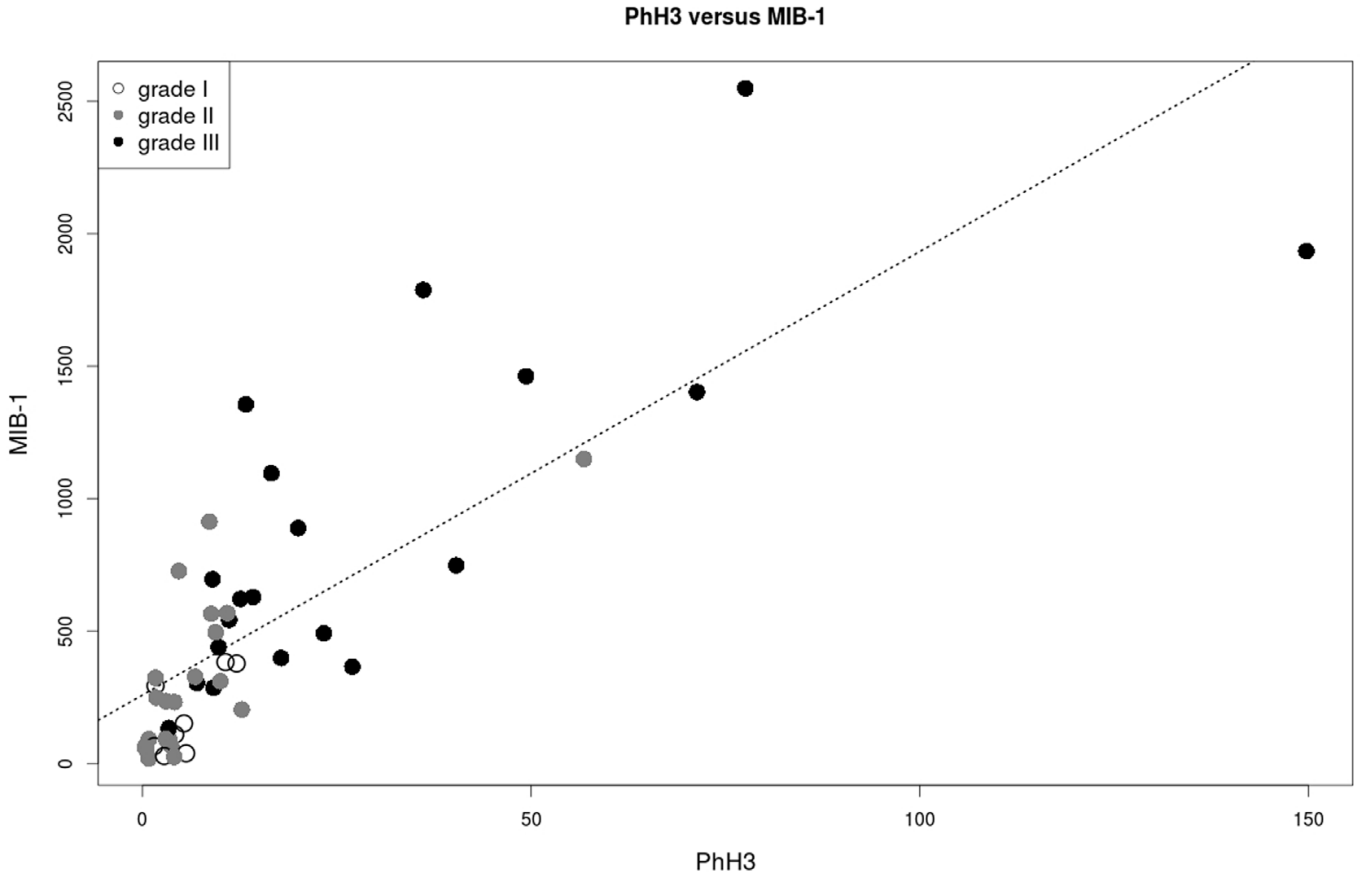
Scatterplot of MIB-1 versus PhH3. Correlation coefficient is 0.79 (r^2^ = 62%). Histological grades are identified with shade of grey (see legend). Tumors found above the regression line have on the average higher MIB-1 counts.

**Figure 3:**
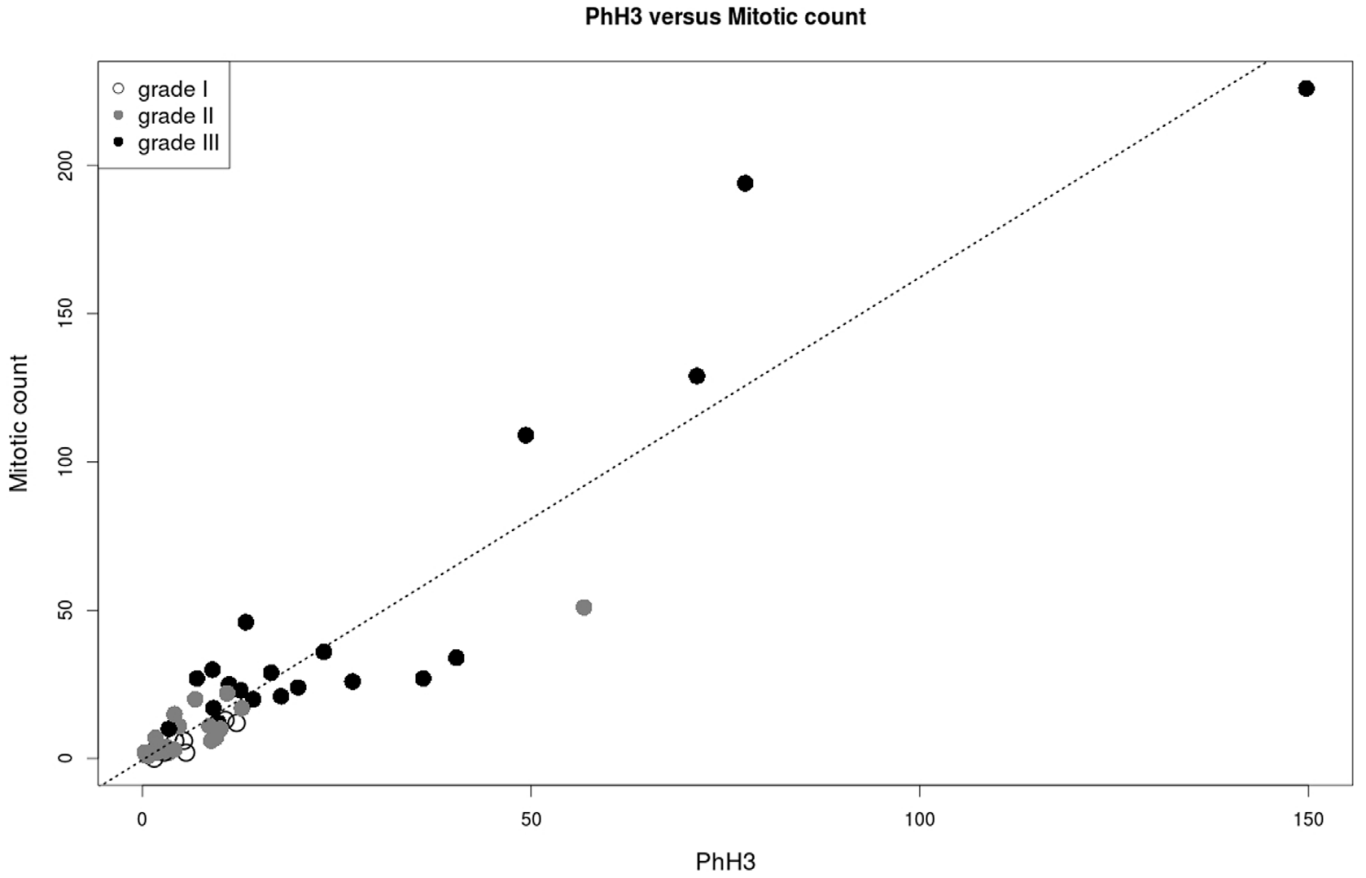
Scatterplot of mitotic count versus PhH3. Correlation coefficient is 0.94 (r^2^ = 88%). Histological grades are identified with shade of grey (see legend).

Forty-nine consecutive cases of invasive breast carcinoma were identified. The clinical characteristics of the patients are summarized in Table 1. Descriptive data regarding the MIB-1 counts, PhH3 counts, mitotic counts and IOD, separated by grade, are summarized in Table 2. Correlations between variables are summarized in Figure 1. The strongest correlation involves mitotic count and PhH3 (r = 0.94). Correlations involving MIB-1 are weaker: r = 0.83 for mitotic count and 0.79 for PhH3. IOD is weakly to moderately correlated with all variables; the strongest correlation is found with MIB-1 (r = 0.37). Figure 2 shows a scatterplot of MIB-1 counts (y axis) versus PhH3 counts (x axis). The best fit linear regression line follows the formula MIB-1 = 16.7×PhH3 + 258. Differentiating the tumor grades, 1 of 8 (12.5%) grade 1, and 5 of 21 (23.8%) grade 2, and 11 of 20 (55.0%) grade 3 tumors lie above the linear regression line. Comparing the low grades (grade 1 and 2) and high grade (grade 3) shows that there is significantly more grade 3 tumors lying above the regression line (p = 0.017). This indicates that high grade breast tumors not only have the highest MIB-1 counts, but also have proportionally higher MIB-1/PhH3 ratios. Figure 3 shows a scatterplot of mitotic count (y axis) against PhH3 (x axis) with associated histological grades and demonstrates the strongest correlation.

**Figure 4:**
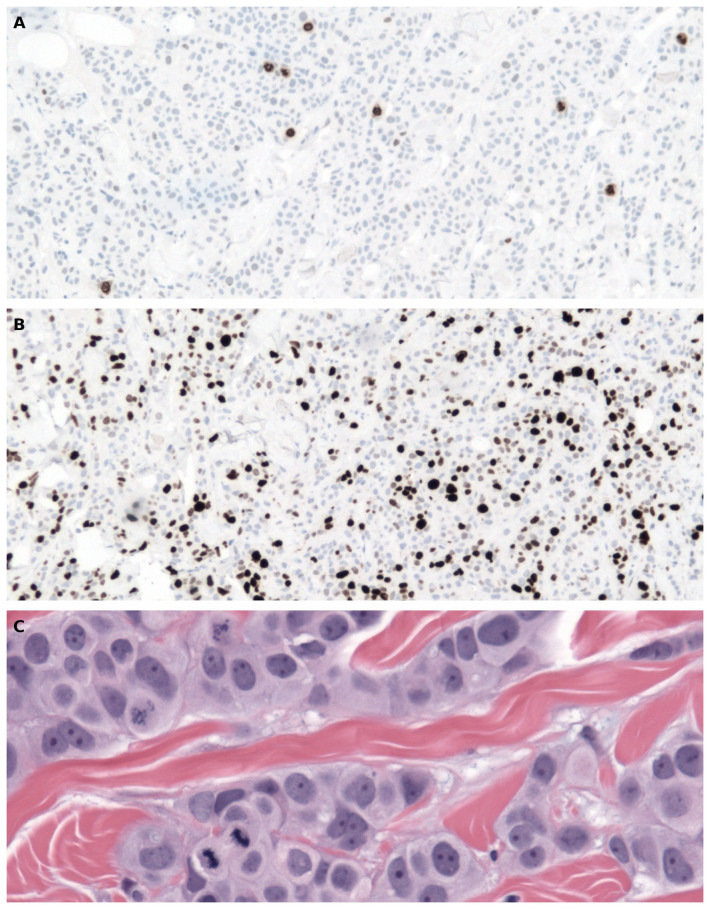
Example of the immunohistochemical staining with PhH3 (*A*, 100 × magnification) and MIB-1 (*B*, 100 × magnification) of the same area of the histology. The MIB-1 and PhH3 counts were performed by the computer using these images. ***C*** shows an area of the haematoxylin and eosin stained section at 400 × magnification. Mitotic figures are counted at this magnification directly from the microscope.

Visual inspection of slides immunostained with PhH3 shows preparations with very high signal-to-noise ratio. Identification of mitoses is thus straightforward especially when compared with mitoses identification in H&E preparations. The same comment applies to the MIB-1 preparation. Examples of images used in PhH3, MIB-1, and mitotic count are shown in Figure 4.

## Discussion

Cell proliferation is an important component in the assessment of many neoplasms. This study evaluated the correlations between MIB-1, PhH3, and mitotic count in a defined area, focusing on invasive breast carcinoma. As expected, a very strong correlation (r = 0.94) between mitotic and PhH3 counts was found. It was as good or better than correlations previously reported (Table 3) because of the attempt in this study to ensure the same tumor areas were assessed in order to neutralize the effect of intratumoral heterogeneity.

As shown in Figure 1, MIB-1 does not correlate as well with PhH3 (r = 0.79) or mitotic score (r = 0.83) as compared with the correlation between PhH3 and mitotic score. A similar trend is observed in the literature (Table 3). Given the optimization of our experimental design to reduce confounders in our comparisons, such as tumor heterogeneity, and the strong correlation between PhH3 and mitotic count found in this study, the discrepancy between MIB-1 and PhH3 or mitotic count would not be explained by experimental error. We suggest, based on review of the literature, that the discrepancy has pathological ground related to cell cycle anomalies found in malignant tumors. A key factor would be the proportion of time a cell spends in the mitotic phase compared to interphase (G1-S-G2 phase). Cell cycle time is known to vary significantly among tissue types, cellular differentiation, and tumors^42^,^43^,^44^.

Because we selected consecutive cases, the distribution of grades matches that found in the breast cancer population: there are fewer grade 1 cases as compared to grade 2 and grade 3^45^. A careful examination of the results of this study indicates that the higher the tumor grade, the more likely that the tumor shows a higher MIB-1 count as compared to PhH3. This is illustrated in Figure 2 which shows that the majority of low grade tumors lie below the linear regression while the majority of grade 3 tumors lie above the line.

This non-linear relationship between grade and the MIB-1 to PhH3 ratio is a phenomenon that can be explained by increased relative durations spent in interphase as compared to mitotic phase in the higher grade tumors. High grade tumors generally have longer cell cycles and, in particular, longer S-phase durations (T_s_). This is because they are more likely to be aneuploid and thus more DNA material must be replicated^46^,^47^,^48^. In a study on DNA ploidy and S-phase fraction (SPF) in breast cancer by Wong et al., histologic grade 3 tumors were more frequently aneuploid and had higher SPF than grade 1 or 2 tumors^46^. While the reason for increased SPF was not studied, a lengthened S-phase, due to increased DNA in aneuploid cells, is one of the most likely explanations. Additionally, a study by Martinez-Arribas et al. found that a rising DNA content as well as increasing percentage of cells in S-phase were both correlated with increasing Ki-67 index^49^. Similar findings are seen in studies regarding T_s_ seen in lung^50^, head and neck^51^, colon^52^,^53^, esophagus^54^ and cervix^54^. These studies showed that aneuploid tumors displayed approximately 25–30% longer T_s_ than diploid tumors^44^. Additional explanations for lengthened interphase in higher grade tumors include increased damage to DNA replication machinery resulting in slower activity by enzymes such as DNA replicase, and increased malfunction of cell cycle control signals which would inhibit transition from interphase into mitosis. Moreover and importantly, while mitotic phase duration lengthens in malignant cells compared to benign cells, when considering only malignant cells, it remains relatively unaffected by ploidy number^55^,^56^. The proportion of time spent in the cell cycle but not in mitotic phase is thus generally higher in higher grade tumors: cell cycle phase durations are consequently decoupled. The latter has an inevitable effect on the number of cells labeled by MIB-1 as compared that PhH3.

**Figure 5:**
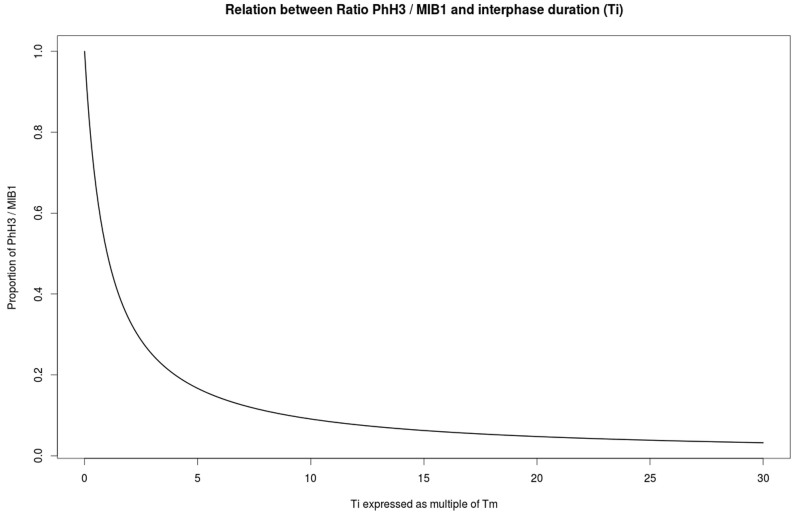
Theoretical relationship between the proportion PhH3/MIB-1 and T_i_ (cell cycle interphase duration) showing decoupled cell cycle phase duration phenomenon among different tumors: as T_i_ increases the proportion PhH3/MIB-1 diminishes. When for instance S phase duration increases in aneuploid tumors, MIB-1 count will be proportionally larger than PhH3 as compared to another tumor with the same MIB-1 count but with a shorter S phase duration. This effect would explain “imperfect” correlation between PhH3 (or mitotic count) versus MIB-1. Also because of lengthened S phase two tumors with identical mitotic count could have different MIB-1 fractions.

Immunohistochemical staining only captures a single time snapshot of the tumor: MIB-1 captures all cells within the cell cycle while PhH3 captures only the cells undergoing mitoses. The consequences of this decoupled cell cycle phase duration can be modeled mathematically. The relationship between the number of cells in mitosis and number of cells in the cell cycle is represented by the equation 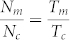 where N_m_ is the number of cells undergoing mitosis at a given time, N_c_ is the number of cells in the cell cycle, T_m_ is the duration of mitosis, and T_c_ is the duration of cell cycle. Given that T_m_ is relatively constant, the proportion of cycling cell undergoing mitosis is inversely proportion to duration of the cell cycle. Also, *T_c_* = *T_m_* + *T_i_* where T_i_ is the duration of interphase. Thus, 
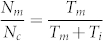
. Since N_m_ can be estimated by the number of cells staining for PhH3, and N_c_ by the number cells staining for MIB-1, 
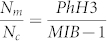
. This ratio is dependent on the duration of interphase T_i_ and is demonstrated in Figure 5. The duration of individual cell cycle phases is thus decoupled due to cell cycle anomalies.

Practically, two different tumors could have the same mitotic or PhH3 counts, but quite different MIB-1 counts. For example, if mitosis was 1/24^th^ the length of a cell cycle, as classically it is described as being 1 hour long with a complete cell cycle taking 24 hours^57^, 4.17% (or 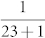) of MIB-1 labeled cells would stain for PhH3. If T_i_ were to increase by 30% from 23 hours to 29.9 hours, then 3.24% (or 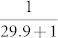) of MIB-1 labeled cells would stain for PhH3, representing a relative decrease in PhH3 staining of 22.3% as compared to MIB-1. While this model is an ideal calculation that does not represent all the variables in a malignancy, the proportion of MIB-1 positive cells expected to stain with PhH3 is relatively congruent with our data shown in the scatterplot (Figure 2) where the associated linear regression, *MIB1* = 16.7 × *PhH3* + 258, indicates that PhH3 counts are approximately 5.9% of the MIB-1 count. Some of the potential additional variables that are not accounted for by our model includes the G0 phase and the fact that MIB-1 does not perfectly stain all cells in the cell cycle, nor does PhH3 staining correspond precisely to mitosis.

IOD results also corroborate with the above discussion. The IOD derived from the H&E sections is an approximation of DNA content. The breast tumors with higher IOD would more likely be aneuploid and would also be associated with disproportionately longer S-phase. Despite poor correlations with all proliferative markers, IOD did show the best correlation with MIB-1, evidence that nuclear content has stronger relationship to overall cell cycle duration than to mitotic duration. It should be noted that the IOD derived from H&E sections is a relatively poor approximate of DNA content because hematoxylin is not stoichiometric to DNA^58^ and the nature of the histological section involves cutting through nuclei.

In the introduction, it was highlighted that proliferative markers currently in clinical use only address the proliferative fraction, P, of the equation 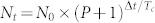. The cell cycle time T_c_ is ignored by most investigators as it is difficult to assess. It is generally suggested that tumors with a higher proliferative fraction would proliferate faster. However, the same proportional variation of P and T_c_ do not have the same impact in term of cell production. For instance, a tumor A with P = 60% should generally proliferate faster than a tumor B with P = 20% (*P_A_*/*P_B_* = 3). However if the former tumor A has a cell cycle time T_c_ = 72 hours and the latter tumor B a cell cycle T_c_ = 24 hours (*T_c_*_(*A*)_/*T_c_*_(*B*)_ = 3), tumor B will produce many more cells than tumor A in the same amount of time. The difference is due to the exponential property of the formula^59^. More than a proliferative or kinetic marker, these “P” markers, and especially MIB-1 since it captures the entire T_c_, are probably better characterized as markers of cell-cycle abnormality.

The previously reported correlations (Table 3), show significant variability between different reports and different tumor types. Part of this variability is explained by intratumoral heterogeneity which are not addressed by their methodologies; none of these studies directly compared proliferative markers on the same tumor areas. For example, in the study with the lowest correlations^20^, mitotic and MIB-1 counts were assessed from random fields in full sections while PhH3 was assessed on tissue micro-array (TMA). Additionally, most of the studies used manual counting methods. Although counting mitoses or cells stained by immunohistochemistry may seem simple and basic, in practice counting mitosis can be difficult^60^ and there is inadequate inter-observer agreement in manual MIB-1 counting^61^,^62^,^63^.

Two of the previous studies focused on breast carcinoma: Bossard^64^ and Zbyteck^2^. It is notable that they gave significantly differing results in their correlation between PhH3 and mitotic counts. It is difficult to compare these two studies as their methodology may differ. Bossard counted consecutive field in the regions of highest activity, while Zbyteck does not specify where they counted. Furthermore, they are both Spearman's correlation coefficients, making them more difficult to compare with our study and with each other. Spearman's correlation coefficient should not be overinterpreted as a measure of the strength of association between two variables, unlike the Pearson correlation coefficient. The low correlation reported by Zbytek may be due to their focus on low grade tumors (117 grade 1 tumors vs. 19 grade 2 and 2 grade 3). Proliferation counts by any method in low grade tumors will be low, differing only by a few counts, thus making them highly susceptible to variations that would affect the rank-order used in Spearman's correlation calculation. Bossard's study, which included 13 cases of each grade, has a grade distribution that is more similar to the current study.

An automated digital image analysis approach was chosen in our study to reduce inter and intra-observer variability. This provided improved reproducibility in the count method across all samples to fulfill our objectives in comparing proliferative markers. Furthermore, we chose to report results as counts per area to increase accuracy of marker comparison. Proliferative markers, most notably MIB-1, are frequently reported as percentage of malignant cells stained (ie. Ki-67 index). While this approach is biologically reasonable, it is difficult to accurately assess the number of malignant cells in a given area (either manually or with image analysis). Because the main goal of this study was to compare proliferative markers among themselves in the same tumor, the choice of denominator (area instead of number of malignant cells) was found to be more reproducible and accurate, and would not affect the math or understanding of the relationships between the markers.

There are limitations to this study. We only focused on invasive breast carcinoma, and even though similar observations were found with other organs (Table 3), they should be studied independently. While computer image analysis has provided increased precision and consistency in its counts as compared to manual counting, our specific count algorithm may differ from algorithms and software used in other studies. This should not be an issue if the counts by the various methods are accurate and well documented.

In conclusion, PhH3 and mitotic count are strongly correlated and could be used interchangeably. PhH3 immunostain showed high signal-to-noise ratio which facilitates assessment of mitotic count either using manual scoring or image analysis. MIB-1 is less correlated with either PhH3 or mitotic count. Review of the literature provided evidence that our results are due to the decoupling of cell cycle phase durations, which we modeled mathematically. Therefore, each proliferative marker addresses a specific aspect of tumor growth assessment, and they will correlate variably when they do not highlight the same phases of the cell cycle. We have also briefly highlighted that the currently used proliferative markers only assess “P”, the fraction of proliferating malignant cells. Other variables, such as cell cycle duration, are difficult to measure and thus ignored, but would be clinical just as important as “P” in measuring tumor growth and aggressiveness. The clinical relevance of our study lies in the clarification of the relationships between the currently used proliferative markers. With the exception of proliferative markers associated with the same phase (e.g. mitotic count and PhH3), each proliferative marker conveys distinct biological information and should be treated separately.

## Methods

### Sample selection and preparation

Consecutive cases of resected invasive breast carcinoma between June 2012–June 2013 with Ki-67 index requests were identified prospectively from the regional tertiary care hospital and cancer center. Government regulations in the province of Alberta (Canada) regarding formalin fixation for breast specimens require ischemic times to be less than 30 minutes. The cases were diagnosed by a breast pathologist and the tissue block containing carcinoma with the region of highest mitotic count, as assessed using the diagnostic hemotoxylin and eosin (H&E) slides, was selected. Two slides were made using four-μm-thick sections for each block. One was stained for Ki-67 (MIB-1, mouse monoclonal, 1/200 dilution, DAKO Corp, Carpinteria, CA). The other slide was stained for PhH3 (rabbit polyclonal, 1/200 dilution, Cell Marque Corp, Rocklin, CA). Antigens were retrieved using heat-induced epitope retrieval with the EDTA based Leica Bond Epitope Retrieval Solution 2 (Leica Microsystems GmbH, Wetzlar, Germany) for 20 minutes at 100 degrees Celsius and pH 9.0. They were processed on the Leica Bond III stainer using the Leica Bond Polymer Refine Detection utilizing a poly-HRP anti-mouse/rabbit IgG reagent that localizes the primary antibody, DAB chromogen, and haematoxylin counter-stain. Each case was reviewed by a breast pathologist for the carcinoma subtype, and the modified Bloom–Richardson–Elston (MBR) grade was re-assessed for consistency and accuracy.

### Data acquisition

Depending on the size of tumor identified in each block, between 4 and 12 fields on each MIB-1 slide were digitized at 10× objective (Nikon Eclipse E600 microscope, 0.25 aperture, Nikon Instruments Inc, Melville, NY) with a QImaging Micropublisher 5.0 RTV camera (QImaging Corp, Surrey, BC) which uses a Sony® ICX282 progressive scan interline CCD producing 24-bit color pictures with a resolution of 2560 × 1920 pixels. A priori background correction^65^ was applied using the ImageJ image processing software (U. S. National Institutes of Health, Bethesda, MD)^66^ following standard procedure. The areas imaged on the MIB-1 slides were precisely marked on the PhH3 and H&E slides. The same areas on each respective PhH3 slide were imaged in the same manner. H&E images for each case were also captured in the same manner, but at high power (40× objective, 0.65 aperture) for nuclear IOD analysis. The images were chosen at random from within the marked locations.

### Counts

Mitotic counts were performed manually directly on the H&E slides using the optical microscope at high power (Nikon Eclipse E600 microscope, 40× objective, 0.65 aperture). Mitoses were counted in ten evenly spaced high power fields (HPF) within the marked locations. MIB-1 and PhH3 counts were performed in the imaged areas with an image-analysis algorithm written with embedded macro ImageJ language. Briefly, the algorithm steps were i) color deconvolution of the initial red-green-blue (RGB) color space into a new color space based on new vectors to isolate DAB stain, ii) “minimum” filter in order to smooth background and signal (DAB), iii) robust automatic threshold selection (creation of binary image), iv) watershed segmentation (to separate touching nuclei), and v) particle count. The average number of particles (MIB-1 or PhH3) per one 10× field was recorded.

### Integrated optical density (IOD)

Using the Wacom Intuos® 4 tablet (Wacom Company Ltd., Tokyo), 15 nuclei were selected at random from the digitized H&E images and their contour were precisely delineated with Intuos pen using the selecting tool available within ImageJ software. Nuclear contours were transformed into vectorial masks and saved as TIFF format files. The latter were submitted to an ImageJ algorithm which computed individual nuclear area and associated IOD of the hemotoxylin nuclear staining. H&E slides used for IOD were four-μm-thick. H&E staining was performed in different batches.

### Statistical analysis

Statistical analysis and figure creation were performed using R language (version 2.14.1, R Foundation, Vienna)^67^. Correlations among all variables were plotted and corresponding Pearson's correlation coefficients (r) were reported. Scatterplots between PhH3 and both MIB-1 and mitotic count were plotted with the grade of each tumor indicated for comparison. Differences among the grades were compared using fisher's exact test for categorical data, and independent samples t-test for continuous data. A p-value of less than or equal to 0.05 was selected as the level of significance in all analyses. Figures illustrating H&E, PhH3 and MIB-1 preparations were created with the FigureJ plugin^68^.

This study was approved by the Alberta Cancer Research Ethics Committee (ACREC, file number 26100).

## Author Contributions

L.L. wrote the main manuscript text. L.L. and G.B. prepared the figures. H.Y. reviewed the pathology and re-graded the tumors. L.L. and G.B. performed the MIB-1, Ki-67, and mitotic counts. L.L. and G.B. performed the data analysis. G.B. performed the IOD measurements. All authors reviewed the manuscript.

## Figures and Tables

**Table 1:** Clinical characteristics of the breast cancer cases (n = 49)

	Number	Percentage
**Mean age**	Mean = 58.2 ± 13.8 years	
<50	10	20.4
> = 50	39	79.6
**Gender**		
Female	48	98.0
Male	1	2.0
**Carcinoma subtype**		
Ductular	40	81.6
Lobular	7	14.3
Other	2	4.0
**MBR Grade**		
I	8	16.3
II	21	42.9
III	20	40.8
**Architectural score**		
1	5	10.2
2	8	16.3
3	36	73.5
**Nuclear score**		
1	1	2.0
2	18	36.7
3	30	61.2
**Mitotic score**		
1	26	53.1
2	7	14.3
3	16	32.7
**ER**		
Positive	41	83.7
Negative	8	16.3
**PR**		
Positive	37	75.5
Negative	12	24.5
**Her2**		
Positive	11	22.4
Negative	38	77.6

**Table 2:** Descriptive statistics of variables MIB-1, PhH3, Mitotic count and IOD. Mean and standard deviation

	Grade 1	Grade 2	Grade 3	P value*	Overall
**Number**	8	21	20		49
**MIB-1 count/image field**	181.10 ± 148.83	324.06 ± 310.39	906.58 ± 647.63	P < 0.0001	538.48 ± 554.30
**PhH3 count/image field**	5.48 ± 3.97	7.42 ± 11.92	30.93 ± 34.80	P = 0.0010	16.70 ± 26.15
**Mitotic count/10 HPF**	5.5 ± 4.78	9.52 ± 11.47	53.25 ± 61.59	P = 0.0003	26.71 ± 45.36
**Nuclear IOD**	1398.50 ± 409.88	1752.94 ± 797.11	2616.78 ± 952.34	P = 0.0002	2047.66 ± 944.16

*Independent t-test, comparing combined grades 1 and 2 with grade 3.

**Table 3:** Correlations (Pearson or Spearman) between mitotic count, PhH3, and MIB-1* in the literature among studies that analyzed PhH3

Author	Site	MIB-1 vs PhH3	PhH3 vs Mitosis	MIB-1 vs Mitosis
Brenner 2003^37^†	Endometrial carcinoma	0.86	0.94	0.86
Bossard 2006^36^‡	Breast carcinoma		0.86	
Kim 2007^21^†	Meningioma	0.77	0.88	0.71
Fukushima 2009^33^‡	Meningioma	0.69	0.66	
Ladstein 2010^20^†	Cutaneous melanoma	0.34	0.15	0.26
Aune 2011^34^‡	Ovarian carcinoma	0.63	0.79	0.77
Hightower 2012^38^‡	Pituitary adenoma	0.11		
Draganova-Tacheva 2013^39^‡	Pancreatic endocrine tumors	0.76		
Idriss 2013^40^‡	Smooth muscle tumors	0.4		0.5
Zbytek 2013^35^‡	Breast carcinoma		0.39 (visual) 0.33 (automated)	

*MIB-1 was reported as Ki-67 index.

^†^Pearson product-moment correlation coefficient.

^‡^Spearman's rank correlation coefficient.
